# Osteoprotegerin Regulates Pancreatic β-Cell Homeostasis upon Microbial Invasion

**DOI:** 10.1371/journal.pone.0146544

**Published:** 2016-01-11

**Authors:** Yukiko Kuroda, Kenta Maruyama, Hideki Fujii, Isamu Sugawara, Shigeru B. H. Ko, Hisataka Yasuda, Hidenori Matsui, Koichi Matsuo

**Affiliations:** 1 Laboratory of Cell and Tissue Biology, Keio University School of Medicine, Tokyo, Japan; 2 Laboratory of Host Defense, WPI Immunology Frontier Research Center (IFReC), Osaka University, Osaka, Japan; 3 Department of Immunology Graduate School of Medicine, University of the Ryukyus, Okinawa, Japan; 4 Mycobacterial Reference Center, The Research Institute of Tuberculosis, Japan Anti-Tuberculosis Association, Tokyo, Japan; 5 Department of Systems Medicine, The Sakaguchi Laboratory, Keio University School of Medicine, Tokyo, Japan; 6 Nagahama Institute for Biochemical Science, Oriental Yeast Co., Shiga, Japan; 7 Kitasato Institute for Life Sciences and Graduate School of Infection Control Sciences, Kitasato University, Tokyo, Japan; Faculté de médecine de Nantes, FRANCE

## Abstract

Osteoprotegerin (OPG), a decoy receptor for receptor activator of NF-κB ligand (RANKL), antagonizes RANKL’s osteoclastogenic function in bone. We previously demonstrated that systemic administration of lipopolysaccharide (LPS) to mice elevates OPG levels and reduces RANKL levels in peripheral blood. Here, we show that mice infected with *Salmonella*, *Staphylococcus*, *Mycobacteria* or influenza virus also show elevated serum OPG levels. We then asked whether OPG upregulation following microbial invasion had an effect outside of bone. To do so, we treated mice with LPS and observed OPG production in pancreas, especially in β-cells of pancreatic islets. Insulin release following LPS administration was enhanced in mice lacking OPG, suggesting that OPG inhibits insulin secretion under acute inflammatory conditions. Consistently, treatment of MIN6 pancreatic β-cells with OPG decreased their insulin secretion following glucose stimulation in the presence of LPS. Finally, our findings suggest that LPS-induced OPG upregulation is mediated in part by activator protein (AP)-1 family transcription factors, particularly Fos proteins. Overall, we report that acute microbial infection elevates serum OPG, which maintains β-cell homeostasis by restricting glucose-stimulated insulin secretion, possibly preventing microbe-induced exhaustion of β-cell secretory capacity.

## Introduction

Osteoprotegerin (OPG, encoded by *Tnfrsf11b*) is a decoy receptor for RANKL, a tumor necrosis factor (TNF) family cytokine (*Tnfsf11*) [1, 2]. RANKL is expressed in osteoblasts and osteocytes and in many other cell types including keratinocytes, activated T cells, and hypothalamic neurons [3]. Its receptor RANK (*Tnfrsf11a*) is expressed in macrophage-osteoclast precursors, dendritic cells, and immature medullary thymic epithelial cells, among others [3]. RANKL induces differentiation of macrophage/osteoclast precursors into bone-resorbing osteoclasts via RANK signaling [4], and OPG suppresses this process. Consistently, mice lacking OPG become osteopenic due to enhanced osteoclastogenesis in all bone tissues, from long bones to auditory ossicles [5–8]. In postmenopausal women, a single dose of a preparation containing Fc-OPG fusion proteins or the human anti-RANKL monoclonal antibody (Denosumab) rapidly and significantly decreases bone turnover for a sustained period [9, 10]. A local increase in the RANKL/OPG ratio is associated with osteoclastogenesis and bone destruction in inflammatory bone disease such as rheumatoid arthritis and periodontal disease [11, 12]. We previously reported increased serum OPG levels accompanied by decreased serum RANKL levels in lipopolysaccharide (LPS)-treated mice [13], but it remains unclear whether OPG upregulation following microbial invasion has any significance outside bone.

Transmembrane Toll-like receptors (TLRs) recognize conserved components of microbes and function to eliminate infection, promote tissue repair, and antagonize tissue injury-induced inflammation [14, 15]. Microbe-specific molecules called pathogen-associated molecular patterns (PAMPs) include: LPS, a component of gram-negative bacteria, bacterial lipoproteins, lipoteichoic acid, fungal zymosan, bacterial flagellin, and nucleic acids. PAMP binding to TLRs activates expression and activity of transcription factors, such as NF-κB and activator protein (AP)-1, which in turn regulates production of inflammatory mediators. AP-1 is a dimeric transcription factor typically composed of a Fos protein (c-Fos, Fosl1, Fosl2 or FosB) and a Jun protein (c-Jun, JunB or JunD) [16].

In the present study, we show that in mice serum OPG levels increase after infection with various microbes. We also show that pancreas and liver are the primary producers of OPG in LPS-treated mouse models, and that achieving maximal serum OPG levels requires activity of AP-1 transcription factors. Most importantly, enhanced OPG production increased cortical bone mineral density and maintained glucose homeostasis under inflammatory conditions. Our results suggest that, in addition to its effects on bone, infection-induced OPG protects pancreatic β-cells by blocking RANKL-RANK signaling.

## Results

### OPG levels in mice increase after microbial infection

**Fig 1:**
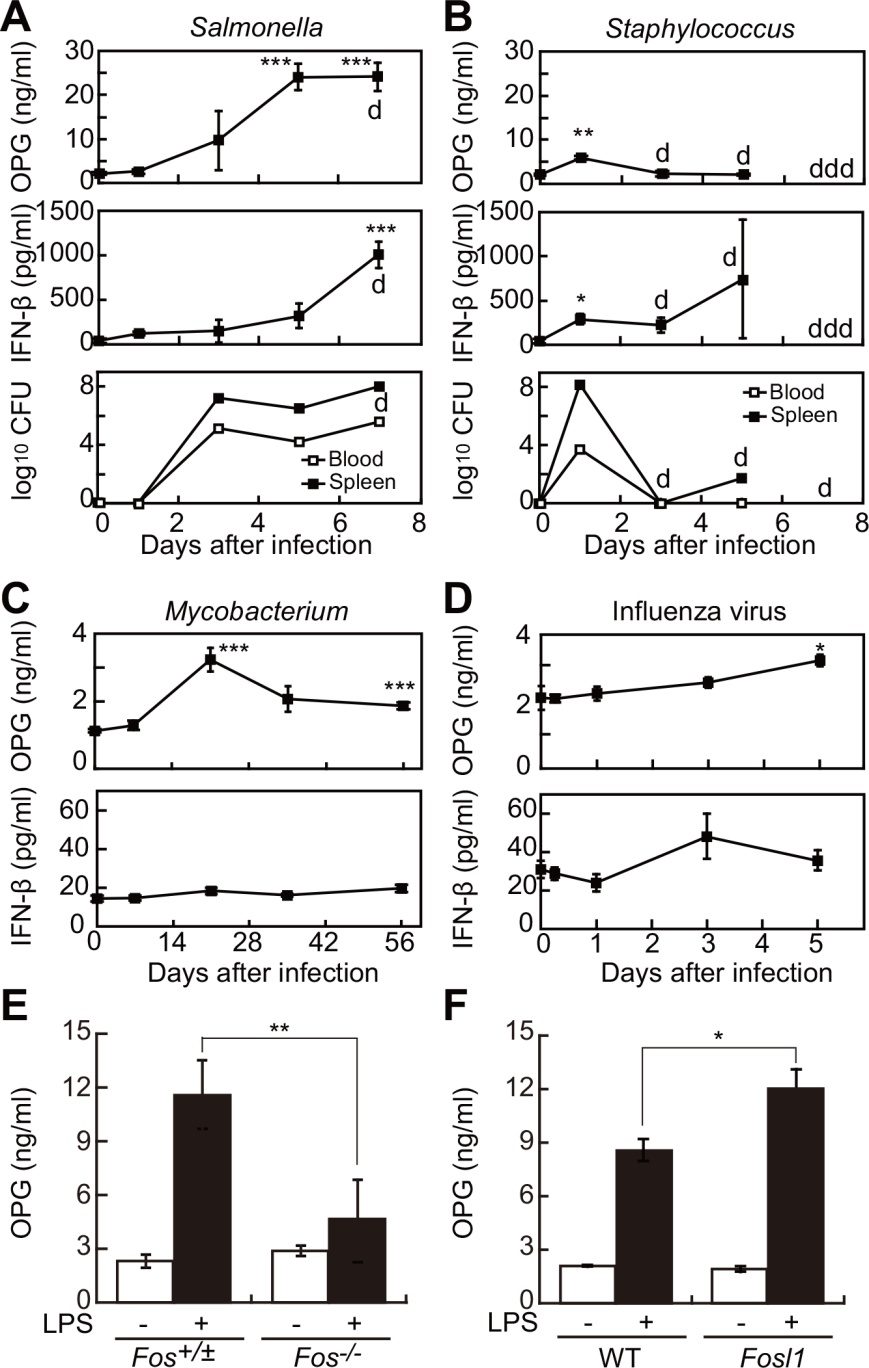
Increased serum OPG levels in mice after microbial infection occurs via Fos family transcription factors. (A, B) Time-dependent elevation of OPG and IFN-β in serum, and colony forming units (CFU) in blood and spleen of 6-week-old C57BL/6J mice infected with *Salmonella enterica* χ3306 (A, n = 4 each point) or *Staphylococcus aureus* 92–1191 (B, n = 4 each point). “d” indicates death of one mouse. “ddd” indicates death of three mice. (C, D) Time-dependent elevation of OPG and IFN-β levels in serum after *Mycobacterium* (C, n = 4 each point) and influenza virus (D, n = 3 each point) infection of 6-week-old C57BL/6J mice. *P < 0.05, **P < 0.01, ***P < 0.005. (E, F) OPG serum levels in LPS-injected mice in c-Fos knockout mice (*Fos*^-/-^) (E) or Fosl1 transgenic mice (*Fosl1*) (F) plus respective littermate controls (n = 3–4). Shown are means ± SD. *P < 0.05, **P < 0.01. LPS (4 μg/g body weight) was administered i.p. to 6-week-old C57BL/6J mice, and blood was collected 12 hr later.

To determine whether serum OPG levels are regulated in response to exposure to various pathogenic microorganisms, we infected mice with *Salmonella enterica* (a Gram-negative bacteria, strain χ3306), *Staphylococcus aureus* (a Gram-positive bacteria), *Mycobacterium tuberculosis*, or influenza virus. *Salmonella* infection gradually increased serum OPG and interferon (IFN)-β levels, preceded by an increase in the number of colony-forming units (CFUs), an indicator of viable bacteria, in blood and spleen over a week (Fig 1A). Similarly, *Staphylococcus* infection transiently increased serum levels of OPG and IFN-β one day after infection, a time point when bacteria were readily detectable in blood and spleen (Fig 1B). Twenty days after *Mycobacterium* infection, serum OPG levels also increased, while influenza virus infection increased OPG serum levels gradually over 5 days (Fig 1C and 1D). These data show that in mice, invasion by a variety of pathogens increases serum OPG levels.

The transcription factors AP-1 and NF-κB are both activated downstream of various TLRs and induce inflammatory responses, including cytokine and chemokine production [17]. Furthermore, mice lacking the prototypical Fos protein, c-Fos, exhibit decreased OPG production relative to littermate controls (Fig 1E), and transgenic mice overexpressing the Fos protein Fosl1 (also known as Fra-1) show enhanced OPG induction relative to controls (Fig 1F). These results strongly suggest that Fos proteins mediate LPS-induced OPG elevation.

### Bone homeostasis in mice after bacterial infection

**Fig 2:**
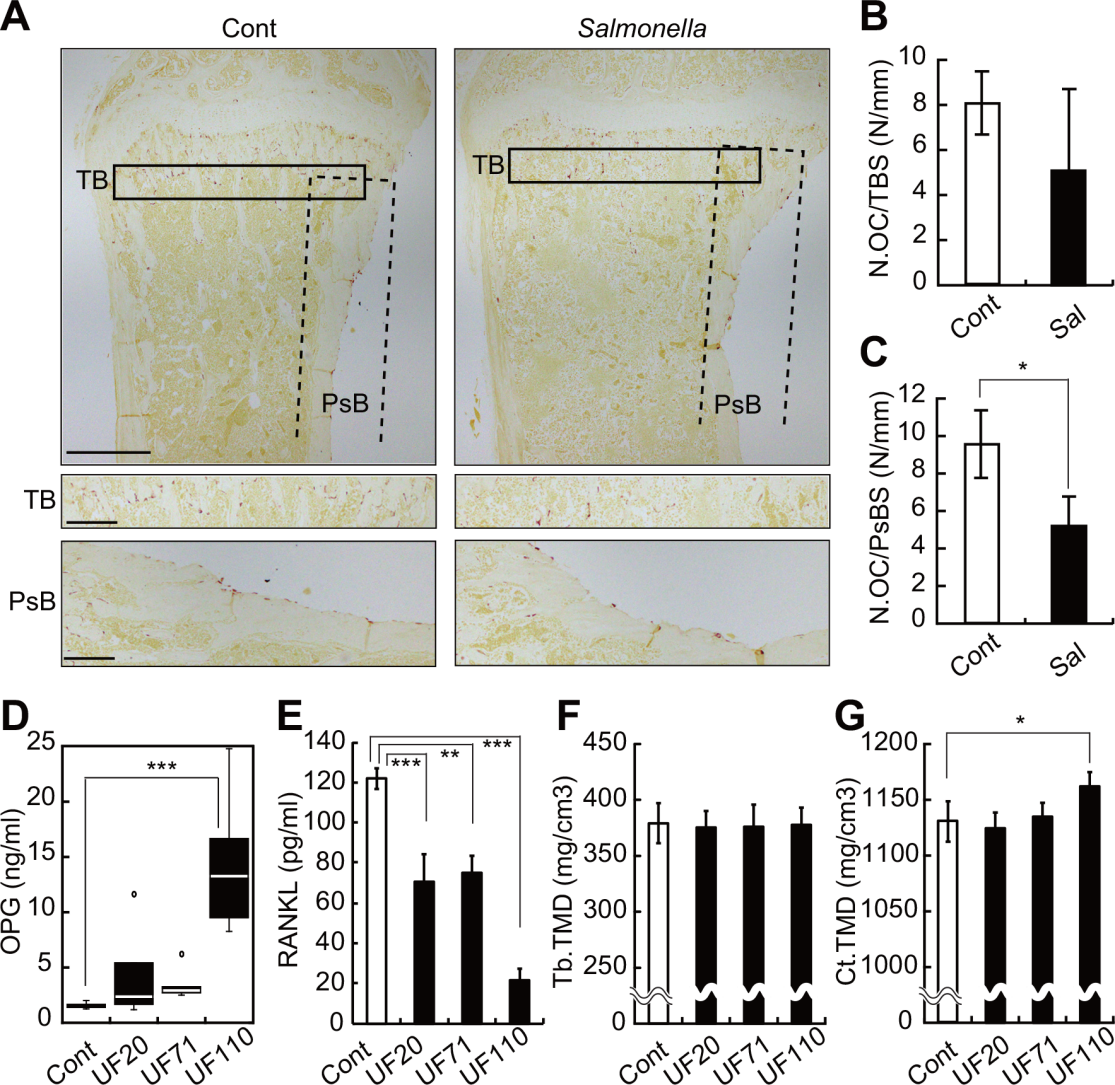
Bone homeostasis in mice after *Salmonella* infection. (A) TRAP staining of tibial sections of BALB/c mice infected for 5 days with *Salmonella enterica*. Cont, uninfected mice; PsB, periosteal bone; TB, trabecular bone. Scale bars in upper panels represent 500 μm, and in middle or lower panels, 200 μm. (B, C) The number of osteoclasts (N.OC) at the trabecular bone surface (TBS) and the periosteal bone surface (PsBS) in tibial sections. Shown are means ± SD. Sal, *Salmonella*-infected mice. (D, E) Serum OPG levels (D) or RANKL levels (E) in mice infected one week with the avirulent *Salmonella enterica* strains UF20, UF71 and UF110 (n = 6 for each group). Open circles indicate outliers. ***P < 0.005. (F, G) Femoral bone tissue mineral density (TMD) in mice infected one week with avirulent *Salmonella*. Tb, trabecular; Ct, cortical. Shown are means ± SD. *P < 0.05 versus each control.

Elevated serum OPG could inhibit osteoclast differentiation and thus perturb bone resorption. To assess this possibility, we first determined the number of osteoclasts by TRAP activity staining, which detects osteoclasts, in both trabecular and periosteum bone in tibiae (Fig 2A) in mice infected with the virulent *Salmonella enterica* strain χ3306 for 5 days, a period during which serum OPG levels were elevated (Fig 1A). The number of osteoclasts significantly decreased at the periosteum after infection, although this trend was not significant on the trabecular surface (Fig 2B and 2C). To assess effects of serum OPG elevation on bone homeostasis independently of *Salmonella* virulence, we undertook similar analysis using the avirulent *Salmonella* strains UF20, UF71 and UF110. Serum OPG levels were most significantly elevated in UF110-infected mice (Fig 2D), whereas serum RANKL levels decreased in mice infected with all strains one week after infection (Fig 2E), indicating that the RANKL/OPG ratio, an index of osteoclastogenic activity, is most significantly decreased in UF110-infected mice. Micro-computed tomography (μCT) revealed that UF110 infection increased tissue mineral density (TMD) of cortical but not trabecular bone by one week after infection (Fig 2F and 2G). These results suggest that bacterial infection-induced OPG elevation inhibits osteoclast differentiation, thereby increasing bone tissue mineral density, particularly in cortical bone.

### LPS-induced OPG production in liver and pancreas

**Fig 3:**
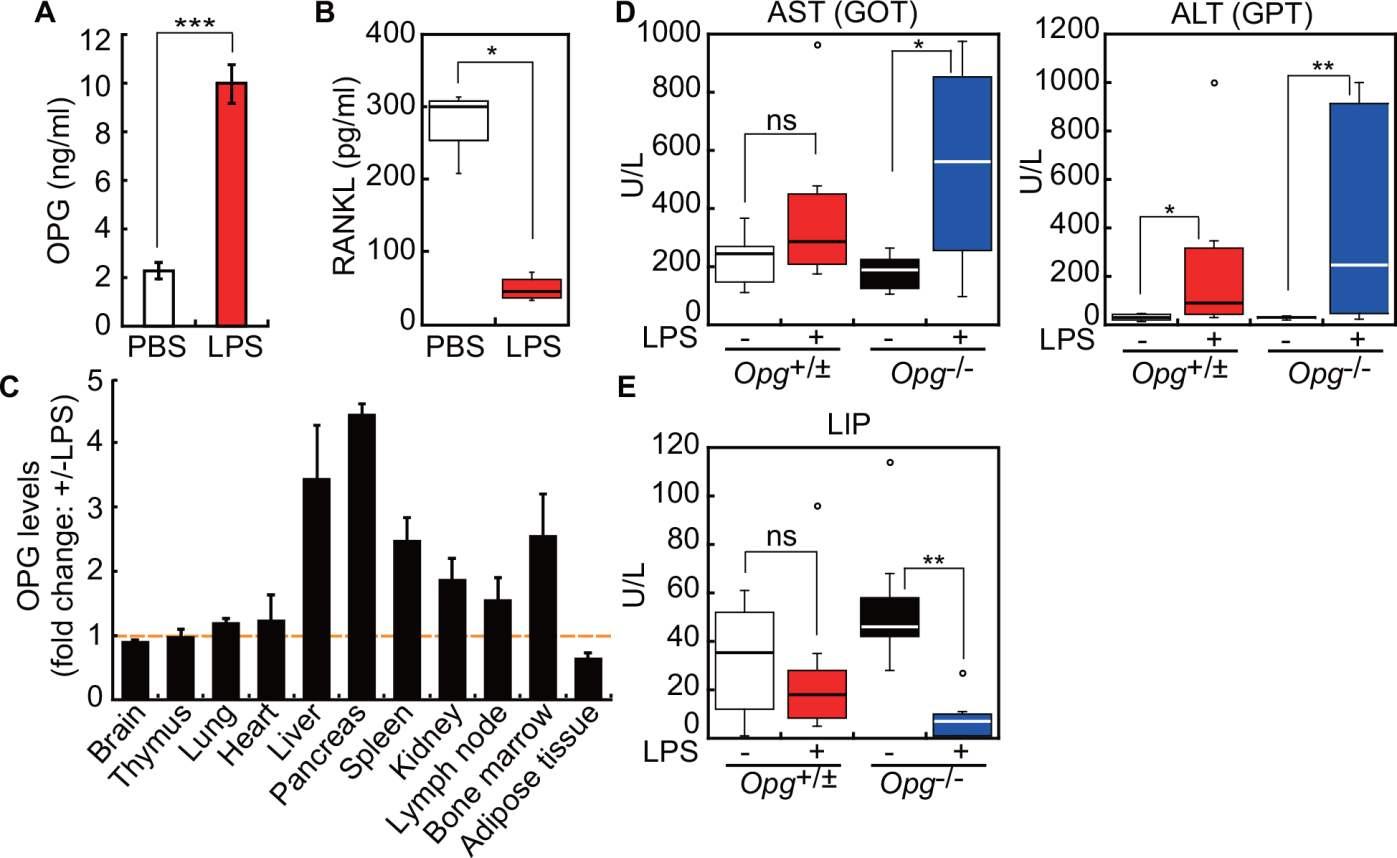
OPG production and biological marker analysis in liver and pancreas following LPS administration. (A, B) Serum OPG (A) or RANKL (B) levels in control PBS-injected or LPS-injected mice (n = 4 for each group). *P < 0.05, ***P < 0.005. (C) Fold-change in OPG protein levels in various organs isolated from LPS-injected relative to PBS-injected control mice (n = 3–5). Protein levels were normalized to total protein. Organs were collected 20 hr after injection of LPS (1 μg/g body weight) i.p. into 6- or 12-week-old C57BL/6J mice. Shown are means ± SEM. (D, E) Biochemical tests of liver and pancreatic function. Box plots show distribution of activities of aspartate transaminase (AST), alanine transaminase (ALT), and lipase (LIP) in serum samples collected from PBS-control or LPS-injected 10-week-old control (*Opg*^+/±^) and OPG knockout (*Opg*^-/-^) mice, 22 hr after injection. Open circles indicate outliers. ns, not significant. *P < 0.05, **P < 0.01.

To determine which organs produce OPG in response to infection, we injected wild-type mice with LPS and measured OPG protein relative to total protein levels in various organs isolated from LPS-injected versus control PBS-injected mice (Fig 3).

Consistently with our previous study [13], serum OPG levels increased (Fig 3A) and serum RANKL levels decreased (Fig 3B) following LPS-treatment relative to PBS-injected controls. OPG production in LPS-injected mice increased >3-fold in liver and pancreas relative to controls (Fig 3C). Elevated serum levels of aspartate transaminase (AST) and alanine transaminase (ALT) indicate possible injury to or inflammation of liver cells [18]. Biochemical tests showed increased AST and ALT activities following LPS treatment (Fig 3D). Differences between control and LPS-treated mice were greater when analysis was conducted in *Opg*^-/-^ rather than wild-type (*Opg*^+/+^) or *Opg* heterozygous (*Opg*^+/-^) littermate mice, suggesting that OPG loss enhances liver cell susceptibility to LPS-induced injury. Fluctuating serum lipase levels are a marker of pancreatic injury. In mouse models of acute pancreatitis induced by cerulein and LPS injection, serum lipase levels increase reaching a maximum at around 10 hr, and return to basal levels by 24 hr [19]. Whereas in LPS-injected control *Opg*^+/±^ mice, lipase activities recovered to control levels within 22 hr, in LPS-injected *Opg*^-/-^ mice, lipase activities 22 hr after injection were even lower than basal levels (Fig 3E), suggesting that the lipase-producing cells were more severely damaged in the absence of OPG. These data suggest that liver and pancreas are more susceptible to inflammatory stimuli in *Opg*^-/-^ relative to littermate controls.

### OPG alters glucose metabolism and insulin secretion

**Fig 4:**
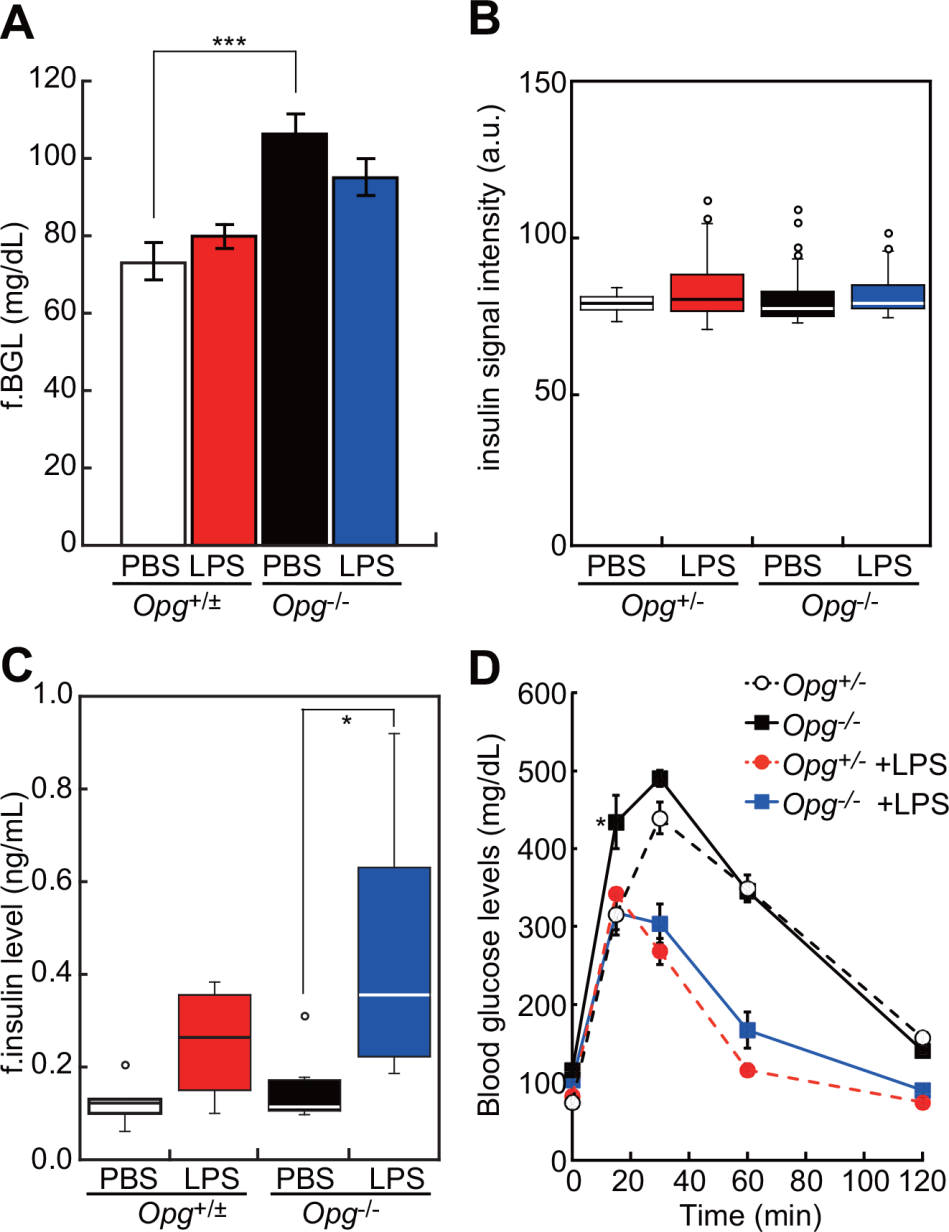
Mice lacking OPG show altered glucose metabolism. (A) Fasting blood glucose levels (f.BGL) in control (*Opg*^+/±^, n = 11, 8) and OPG knockout (*Opg*^-/-^, n = 14, 9) mice 16 hr after injection of PBS or LPS. Shown are means ± SEM. ***P < 0.005. (B) Insulin signal intensity, calculated by insulin immunosignal intensity of cross-sections of pancreata of *Opg*^+/-^ and *Opg*^-/-^ mice 16 hr after injection of PBS or LPS (31<n< 46, each group). Open circles indicate outliers. (C) Fasting (f.) insulin levels in *Opg*^+/±^ (n = 5, 4) and *Opg*^-/-^ (n = 8, 8) mice 16 hr after injection of PBS or LPS. Open circles indicate outliers. *P < 0.05. (D) Intraperitoneal glucose tolerance tests (IPGTT) of PBS- or LPS-injected *Opg*^+/-^ (n = 5, 4) or *Opg*^-/-^ (n = 6, 5) mice. Shown are means ± SEM. *P < 0.05. Blood glucose levels were measured at different time points, as indicated.

The response of liver and pancreas to LPS injection, particularly that seen in *Opg*^-/-^ mice, prompted us to assess OPG function in glucose metabolism following LPS stimulation. To do so, we first examined blood glucose levels (BGLs) in *Opg*^-/-^ relative to littermate control mice. Interestingly, fasting BGLs observed in *Opg*^-/-^ mice were higher than those seen in littermate controls in the absence of LPS treatment (Fig 4A). Analysis of BGL levels in *Opg*^-/-^ mice 16 hr after LPS injection indicated a decrease in those abnormally high levels (Fig 4A). In fasting conditions, insulin production in pancreatic islets did not differ significantly between genotypes, or between without or with LPS injection, based on analysis of insulin immunofluorescence intensity (Fig 4B). LPS injection did, however, significantly elevate serum insulin levels in *Opg*^-/-^ mice, an effect not seen in littermate controls (Fig 4C). Following an intraperitoneal glucose tolerance test (IPGTT), we observed that BGLs increased more rapidly in *Opg*^-/-^ relative to littermate controls after glucose administration, although these changes were indistinguishable in *Opg*^-/-^ and littermate control mice following LPS pretreatment (Fig 4D). These results suggest that insulin secretion increases in the absence of OPG under LPS stimulation and that OPG inhibits insulin secretion under acute inflammatory conditions.

### OPG and RANKL-RANK signaling in β-cells regulates glucose-stimulated insulin secretion

**Fig 5:**
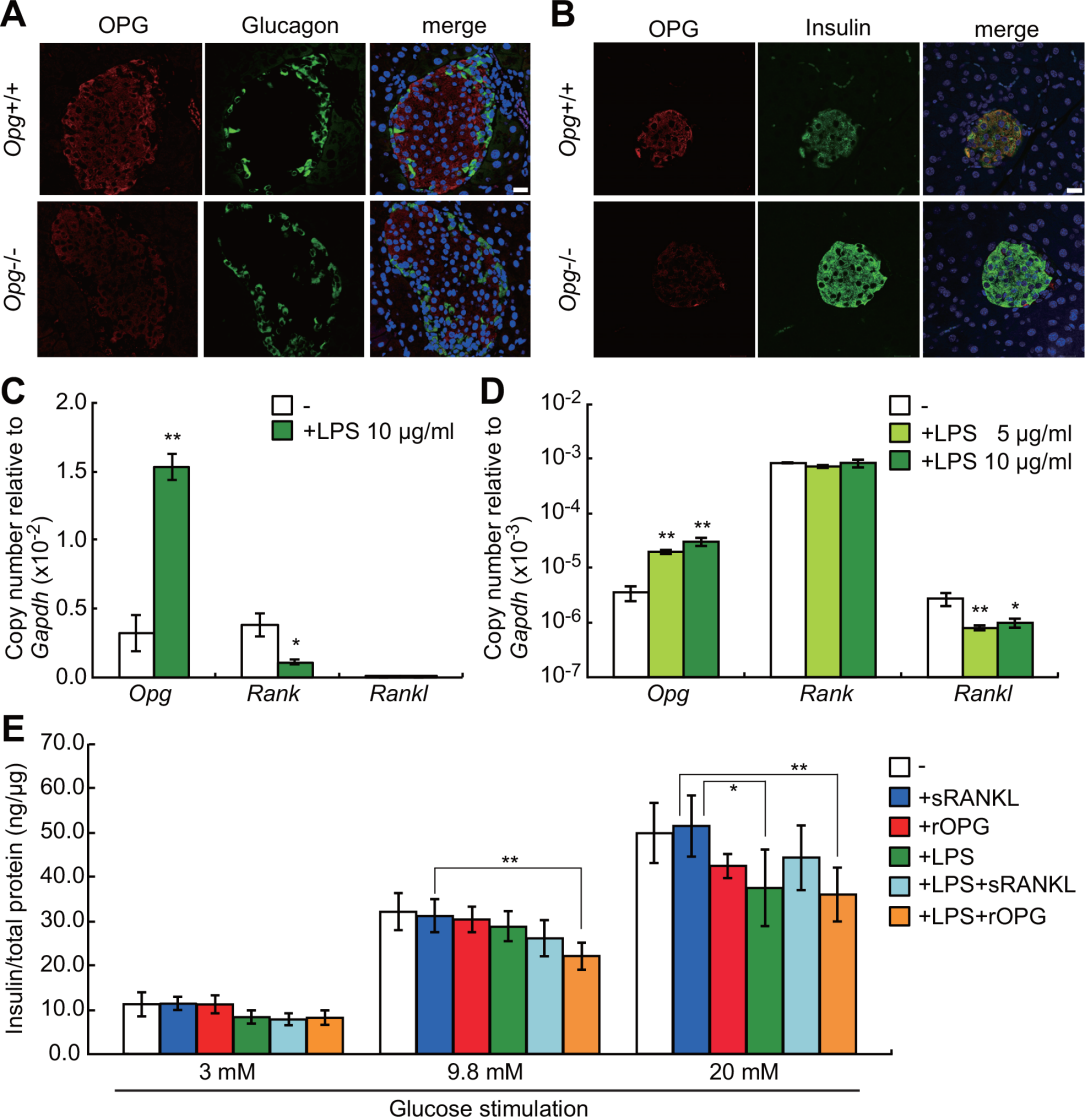
OPG expression in mouse β-cells and effect of OPG on glucose-stimulated insulin secretion. (A, B) Immunofluorescence analysis showing localization of OPG protein (red) with glucagon (green) (A) or insulin (green) (B) in pancreatic islets. OPG is predominantly expressed in β-cells based on co-localization with insulin. Scale bars, 20 μm. (C) Expression of *Opg*, *Rank*, and *Rankl* transcripts in isolated islets treated without or with LPS as measured by qPCR (n = 3–6). (D) Effect of LPS treatment on *Opg*, *Rank* and *Rankl* expression in MIN6 cells (n = 3). (E) Insulin secretion by MIN6 cells. Cells were untreated (n = 6) or treated with 100 ng/ml soluble RANKL (sRANKL, n = 6), 100 ng/ml recombinant OPG (rOPG, n = 6), 10 μg/ml LPS (n = 6), both LPS and sRANKL (n = 6), or both LPS and rOPG (n = 6), and then stimulated with 3, 9.8, or 20 mM glucose. Levels of secreted insulin were normalized to total cell protein. Shown are means ± SD. *P < 0.05, **P < 0.01.

In order to determine which cells express OPG, we undertook immunofluorescence analysis with antibodies to OPG, insulin and glucagon in tissue sections from pancreas of wild-type mice. OPG was localized to insulin-positive β-cells but not to glucacon-positive α-cells in mouse pancreas (Fig 5A and 5B). *Opg* and *Rank* transcripts, but not *Rankl* transcripts, were expressed in islets isolated from mouse pancreas (Fig 5C, open bars). When we treated isolated islets with LPS, *Opg* expression increased, while *Rank* expression decreased (Fig 5C, green bars). Similarly, in the mouse pancreatic MIN6 β-cell line, *Opg* expression, but not that of *Rank*, increased, and *Rankl* expression remained low following LPS treatment (Fig 5D). These results suggest that LPS treatment attenuates RANK signaling in β-cells, thereby regulating insulin secretion. Therefore, we assessed insulin secretion from MIN6 cells treated with various combinations of LPS, soluble RANKL (sRANKL), or recombinant OPG (rOPG) (Fig 5E). Following stimulation with 3 mM glucose, levels of insulin secreted from MIN6 cells were comparable under any condition tested (Fig 5E, left). At 9.8 mM glucose, decreased insulin secretion was seen in cells treated with LPS+rOPG (Fig 5E, middle). At 20 mM glucose, cells treated with LPS alone exhibited decreased insulin secretion. Whereas rOPG addition did not further decrease insulin secretion, sRANKL addition restored insulin secretion in the presence of LPS (Fig 5E, right). These data indicate that insulin secretion is decreased significantly by OPG in the presence of LPS.

## Discussion

Overall, our data establish that in mice infection with a wide range of pathogens, from bacteria to viruses, elevates serum OPG levels (Fig 1). This finding is consistent with previous observations that not only LPS but also synthetic lipopeptides induce serum OPG in mice [13, 20]. Consistently, in humans infection with cytomegalovirus [21], hepatitis C virus [22], or cryptosporidium parasites elevates OPG levels [23]. Data presented here suggest that AP-1 family transcription factors (dimers of Fos and Jun proteins) regulate LPS-induced OPG elevation. This activity may be mediated by an AP-1-like binding site (TGACTGA) in the *Opg* promoter [24]. Furthermore, elevation of OPG by infection had functional consequences: experiments employing the avirulent Salmonella strain UF110 showed that cortical tissue mineral density of infected mice increased by one week after infection with decreased osteoclast numbers (Fig 2). Among avirulent Salmonella strains, only UF110 infection (not infection with UF20 or UF71) increased both serum OPG levels and cortical tissue mineral density. These avrulent strains carry deletion mutations in different virulence genes [25],[26],[27]. The fact that UF110 (not UF20 or UF71) can establish persistent infection [26] likely explains the observed high serum OPG induction and increased cortical tissue mineral density seen in UF110-infected mice.

Elevation of serum OPG in response to infection seems counterintuitive, given the body of literature reporting that local administration of LPS to mouse calvaria activates osteoclastic bone resorption and results in bone loss [28–30]. Indeed, LPS can stimulate osteoclastogenesis via TLR4 signaling through production of inflammatory cytokines such as TNF-α and interleukin (IL)-6 in various cells, through RANKL-upregulation in osteoblasts, and by enhancing survival of mature osteoclasts [31, 32]. On the other hand, TLR-stimulation reportedly inhibits osteoclastogenesis *in vitro*, although whether OPG functions in this process remains unclear [33–35].

Bacterial or viral infection can initiate pancreatic β-cell damage and enhance development of autoimmune diabetes [36]. Furthermore, TLR4/CD14 signaling can induce hepatic insulin resistance [37, 38]. Our study demonstrates that pancreatic β-cells produce OPG in response to LPS exposure. First, pancreata isolated from LPS-injected mice showed a greater than four-fold elevation in OPG protein (Fig 3). Second, pancreatic β-cells were positive for OPG immunostaining (Fig 5). Third, LPS induced *Opg* mRNA expression in isolated pancreatic islets and β-cell line MIN6 (Fig 5). Consistently, Schrader *et al* reported that the rat β-cell line INS-1E and human primary pancreatic islets express *Opg* mRNA and secrete OPG protein, and that both are upregulated by IL-1β and TNF-α [39].

Since LPS induces OPG production by liver and pancreas, we asked whether liver or pancreatic function was altered in OPG-deficient mice. We observed that following LPS treatment, levels of hepatic AST and ALT were increased and pancreatic lipase were decreased more dramatically in OPG-deficient relative to littermate control mice (Fig 3), suggesting that OPG maintains liver and pancreatic homeostasis. We therefore determined whether pancreatic β-cell function and blood glucose homeostasis were perturbed in mice lacking OPG. We found that fasting BGL was higher in *Opg*^-/-^ than in wild-type mice, suggesting that OPG contributes to glucose homeostasis (Fig 4A). Following LPS administration, *Opg*^-/-^ mice showed significantly elevated fasting insulin levels, an effect not seen in littermate controls, suggesting that OPG suppresses insulin secretion induced by LPS (Fig 4C). Stress-hyperglycemia in sepsis [40] can be explained, at least in part, by LPS-induced OPG production in β-cells. Consistently, in MIN6 cells, rOPG treatment suppressed glucose-induced insulin secretion in the presence of LPS was abolished following addition of sRANKL (Fig 5E). The RANKL/RANK/OPG system is implicated in regulation of glucose metabolism [41]. Systemic or hepatic blockage of RANKL signaling in mice significantly improves hepatic insulin sensitivity and promotes normalization of BGL in type 2 diabetes mellitus mouse models [42]. OPG and Denosumab reportedly stimulate β-cell proliferation by blocking RANKL signaling [43], and rOPG-treated mice show decreased insulin secretion in β-cells [44]. These and our studies suggest that OPG mediates multiple protective activities in pancreatic islets.

**Fig 6:**
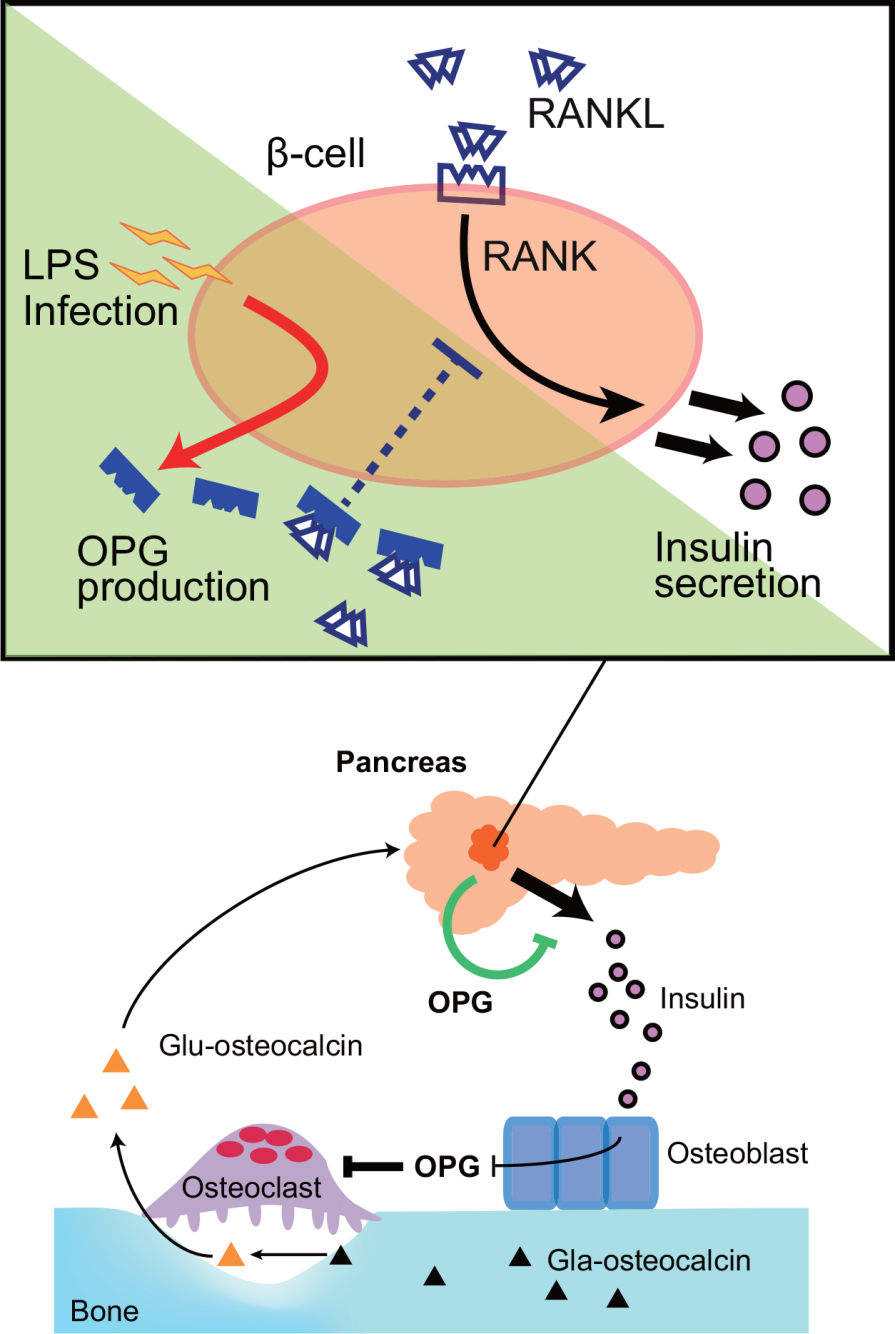
OPG inhibits insulin secretion from β-cells under inflammatory conditions. When β-cells are exposed to inflammatory stimuli, they secrete OPG, which blocks RANKL-RANK signaling. Both osteoblast- and β-cell-derived OPG negatively regulates insulin secretion. Lower panel was adopted from Wei and Karsenty (2015) [45]. Glu, undercarboxylated. Gla, carboxylated.

Finally, OPG is known to reduce serum undercarboxylated (Glu)-osteocalcin levels by suppressing osteoclastic bone resorption, and Glu-osteocalcin acts as a hormone to stimulate insulin secretion in β-cells [45]. Our study demonstrates that OPG acts not only through effects on osteoclasts but also via direct effects on pancreatic β-cells to down-regulate insulin secretion (Fig 6).

## Materials and Methods

### Ethics statement

All mice were maintained under specific pathogen-free conditions, and experiments were performed in accordance with the Institutional Guidelines on Animal Experimentation at Keio University, the Research Institute of Tuberculosis, or Kitasato University. The protocols were approved by the Committee on the Ethics of Animal Experiments of Kitasato University (approval number: 10–036) and by the Keio University Institutional Animal Care and Use Committee (approval number: 09221). Tissue or blood samples were collected after cervical dislocation performed under sevoflurane anesthesia or carbon dioxide asphyxiation, and all efforts were made to minimize animal suffering.

### Mice

C57BL/6J mice were purchased from *Charles River Japan or CLEA Japan Inc*. *Fos*^*-/-*^ mice [46] were on a mixed 129 and C57BL/6J background. Fra-1 mice [47] were backcrossed to C57BL/6J mice for more than seven generations, and mice lacking OPG (*Opg*^-/-^) [5] were bred on a C57BL/6J background.

### Microbial strains and infection

For *Salmonella* infection, *S*. *enterica* serovar Typhimurium SR-11 strains χ3306 (virulent strain) [48], UF20 (avirulent strain; *aroA*::Tn*10*) [25], UF71 (avirulent strain; *phoP*::*aph*-ΔTer) [26], and UF110 (avirulent strain; spvRABCD::tet) [27] were grown in L-broth (Difco) at 37°C with aeration to mid-log phase and then used to infect mice. After 6 hr without food or water, 6–7 week-old female C57BL/6J or BALB/c mice were orally administered 50 μl 10% (w/v) sodium bicarbonate shortly before oral inoculation with 5 x 10^8^ CFU salmonellae suspended in 20 μl PBS, pH7.4, containing 0.01% (w/v) gelatin (buffered saline with gelatin, BSG), as described [49]. Infection was then allowed to proceed for various time periods. Blood was then collected by heart puncture. Spleen was removed and homogenized in BSG. All samples were diluted with BSG and plated on L-agar (Difco)-containing plates with relevant antibiotics in order to assess CFU.

For *Staphylococcus* infection, the methicillin-resistant *S*. *aureus* (MRSA) strain 92–1191 [50] was grown in Brain-Heart Infusion (BHI) broth (Difco) at 35°C overnight with aeration. For infection studies, 6–7 week-old female C57BL/6J mice were intraperitonially inoculated with 2 x 10^8^ CFU staphylococci suspended in a 100 μl of BSG containing 10% (w/v) mucin (Difco). Blood and spleen samples obtained were plated on BHI-agar (Difco).

For *Mycobacterium tuberculosis* infection, 6–7 week-old female C57BL/6J mice were administered microbes using an inhalation exposure system (model 099CA4212; Glas-Col, Inc.), as described [51]. Briefly, a nebulizer compartment was filled with 5 x 10^6^ CFU/5 ml of the virulent Kurono strain (ATCC 358121) to provide uptake of 200–500 viable bacilli by the lungs in 90 min. Lungs and spleens were retrieved from mice at 1, 3, 5, 7, and 12 weeks after infection (three mice per time point). Where indicated, 3.36 x 10^8^ CFU/mouse *Mycobacteria* was injected i.p.

For influenza virus infection, the A/Puerto Rico/8/34 (A/PR8; H1N1) strain was prepared as described [52]. For infection studies, 6–7 week-old female C57BL/6J mice were anesthetized and infected by intranasal administration of 20 μl PBS containing a virus suspension with 2,000 plaque-forming units (PFU) into each nostril.

### LPS treatment

LPS (*S*. *Minnesota* Re595, Sigma) was administered intraperitonially at 4 μg/g body weight to 6-week-old female C57BL/6J mice, and blood was collected 12 hr later to measure serum OPG levels. LPS was injected at 1 μg/g body weight into 6-week-old female C57BL/6J mice or 10–12 week-old male *Opg*±/± mice to measure tissue OPG levels, blood glucose levels, or serum insulin levels, and specimens were collected 16–22 hr after injection.

### ELISA and biochemical tests

Respective protein levels in mouse sera and culture media were evaluated using ELISA kits for OPG (R&D Systems), IFN-β (BD PharMingen), or insulin (Morinaga Institute of Biological Science). Blood glucose levels were measured using Accu-Chek Aviva Nano (Roche Diagnostics). Enzymatic activities of aspartate aminotransferase (AST), alanine aminotransferase (ALT), and lipase were measured using Fuji Dry-chem 3500i (Fuji film).

### MIN6 cells

A method to assay insulin secretion *in vitro* using MIN6 cells [53] has been described [54]. Briefly, MIN6 cells were maintained in Dulbecco’s modified Eagle’s medium containing 4.5 g/L D-glucose (Wako), 10% fetal bovine serum, 0.1 mM 2-mercaptoethanol, 100 units/ml penicillin, and 0.05 mg/ml streptomycin in humidified 5% CO_2_ at 37°C. Cells were replated and cultured for 3 days, and then treated with 10 μg/ml LPS, 100 ng/ml recombinant soluble RANKL (R&D systems), or 100 ng/ml recombinant OPG (R&D systems) as indicated. After additional culture for 24 hr, cells were starved in HEPES-balanced Krebs-Ringer bicarbonate (HKRB) buffer with 3 mM glucose (Cosmobio) for 30 min and then incubated 1 hr in HKRB buffer containing 3, 9.8, or 20 mM D-glucose. Culture supernatants were collected for insulin measurement. Cell lysates were prepared with RIPA buffer to measure protein using the BCA Protein Assay Reagent (Thermo Fisher Scientific).

### Quantitative RT-PCR (qRT-PCR)

Total RNAs from pancreatic islets or MIN6 cells were extracted using ISOGEN (Nippon Gene). cDNA was synthesized from total RNA using random hexamers (Qiagen) with PrimeScript (Takara Bio). Real-time PCR was performed on an ABI 7500 Fast (Life Technologies) apparatus. TaqMan PCR was performed using Premix Ex Taq (Takara Bio). TaqMan probes specific for *Tnfrsf11b* (Mm00435452_m1), *Tnfsf11* (Mm00441908_m1), *Tnfrsf11* (Mm00437132_m1), and *Gapdh* (Mm03302249_g1, Mm99999915_g1) were purchased from TaqMan Assays-on-Demand Gene Expression Products (Life Technologies). mRNA levels were quantified using a standard curve generated with serially-diluted plasmids containing the PCR amplicon and normalized to Gapdh expression.

### Immunohistochemistry

Isolated mouse pancreata were fixed using Mildform (Wako) overnight at 4°C and embedded in paraffin (Sakura Finetek Japan). Sections of 4 μm were treated with 0.2% Triton-X100/PBS for 5 min and incubated with boiled 0.01 M citrate buffer, pH 6.0, for 20 min. Prior to application of primary antibody, sections were treated with Blocking Solution A from the Histofine MOUSESTAIN kit (Nichirei). Primary antibodies, including anti-OPG rabbit polyclonal antibody at 5.0 μg/ml (ab73400, abcam), anti-insulin mouse monoclonal antibody IN-05 at 1.0 μg/ml (ab7760, abcam), and anti-glucagon mouse monoclonal antibody K79bB10 at 4.4 μg/ml (ab10988, abcam) were applied overnight at 4°C. After washing with PBS, cells were stained with Alexa 488-conjugated goat anti-mouse IgG or Alexa 594-conjugated goat anti-rabbit IgG (Molecular Probes) for 1 hr at room temperature. After washing, sections were mounted with Vectashield mounting medium with DAPI (Vector Laboratories) and observed under an LSM710 confocal fluorescence microscope (Carl Zeiss) to determine signal localization or under a DMi6000B microscope (Leica) to measure signal intensity. The average signal intensity was measured by freehand region of interest (ROI) using Tissue Studio (Definiens) software to quantify insulin expression levels in pancreatic β-cells.

### TRAP staining

Mouse tibia were isolated and fixed in 4% paraformaldehyde (PFA) overnight and then decalcified in 0.5 M EDTA at 4°C for 2 weeks. Samples were embedded in paraffin and sectioned at 7 μm. Sections were also stained for tartrate resistant acid phosphatase (TRAP) activity using an Acid Phosphatase, Leukocyte (TRAP) Kit (387A, Sigma-Aldrich) with 20 mM tartrate.

### Bone tissue mineral density

Isolated bones were air-dried and imaged with a μCT scanner (R_mCT; Rigaku, Tokyo, Japan), and bone tissue mineral densities (TMD) of trabecular and cortical bone between 0.5 to 1.5 mm proximal to the femoral distal growth plate were quantified using the TRI/3D-BON system (Ratoc System Enginering, Tokyo, Japan).

### Statistical analysis

Statistical analyses were performed using SPSS ver. 22 software (SPSS Co., Chicago, IL, USA). Data from normal distribution and equal variances were expressed as means ± SD or means ± SEM. Significant differences between control and experimental groups were evaluated using Student's *t*-test or one-way ANOVA with a post-hoc Tukey test. Non-normally distributed data were graphed by box plot using KaleidaGraph (Synergy Sofware Inc., Reading, PA, USA). Data were analyzed using the Kruskal-Wallis test. *P*< 0.05 was considered statistically significant.
